# The complete mitochondrial genome of the endangered freshwater fish *Kichulchoia brevifasciata* in Korea

**DOI:** 10.1080/23802359.2021.1886015

**Published:** 2021-03-15

**Authors:** Kang-Rae Kim, Mu-Sung Sung, Yong Hwi Kim, Jong Yeon Park, In-Chul Bang

**Affiliations:** Department of Life Science and Biotechnology, Soonchunhyang University, Asan, Republic of Korea

**Keywords:** Complete mitochondrial genome, Cobitidae, *Kichulchoia brevifasciata*, endangered fish

## Abstract

*Kichulchoia brevifasciata* is an endangered fish that is distributed across the Goheung Peninsula, South Korea. This is the first report of the complete mitochondrial genome of *K. brevifasciata*, which consists of 16,646 bp with 13 protein-coding genes, 2 ribosomal RNA genes, 22 transfer RNA genes, and a control region (D-loop). The overall base composition of the complete genome is A (29.57%), T (28.08%), G (16.54%), and C (25.80%), with a high A + T content of 57.65%. Phylogenetic analysis showed that *K. brevifasciata* was most closely related to *K. multifasciatas*.

*Kichulchoia brevifasciata* is a dwarf loach with a total length of less than 70 mm (Ko and Bang 2014). It is an endemic species of Korea and belongs to the Cobitidae family. *K. brevifasciata* inhabits rivers with high flow rates and riverbeds made of gravel, and its distribution is limited to the Goheung Peninsula, Geogeumdo and Geumodo Island (Kim et al. 2012). The Republic of Korea Ministry of Environment designated *K. brevifasciata* as an endangered wildlife class II species due to environmental pollution and river development, but its habitat continued to decline and it was upgraded to endangered wildlife class I in 2017 (Kim and Park 2012). In this study, we report the complete mitogenome of *K. brevifasciata*, which will aid conservation of this species.

*K. brevifasciata* was collected from the Sinpyeongcheon Stream under a collection permit issued by the Ministry of Environment in Korea. *K. brevifasciata* genomic DNA was extracted from the caudal fin using the Genomic DNA Prep Kit (Biofact, Korea), and the extracted genomic DNA was stored at Soonchunhyang University. The complete mitochondrial DNA sequence was sequenced using the MGISEQ-2000 protocol (MGI, China) according to the MGI Easy DNA Library Prep Kit (MGI) manual. The raw *K. brevifasciata* sequencing data were assembled using Geneious (ver. 11.0.3) software and annotated using the MITOS web server (Bernt et al. 2013). The complete mitochondrial DNA sequence of *K. brevifasciata* was deposited in GenBank with accession number MW092826.

The mitochondrial genome of *K. brevifasciata* consists of 16,646 bp harboring 13 protein-coding genes (PCGs), 2 ribosomal RNA (rRNA) genes, 22 transfer RNA (tRNA) genes, and a control region (D-loop). Of the 13 PGCs, only *CO1* starts with GTG, and the other 12 start with ATG. Three PCGs (*CO3*, *Cytb*, and *ND3*) terminate with an incomplete stop codon T, and two (*ND2* and *ND4*) with TA, and eight (*CO1*, *ATP6*, *ATP8*, *ND1*, *ND4L*, *ND5*, *ND6*, and *CO2*) with a complete TAA or TAG. The overall base composition of the *K. brevifasciata* genome is A (29.57%), T (28.08%), G (16.54%), and C (25.80%), with a high A + T content of 57.65%.

A phylogenetic tree was constructed using the Neighbor-Joining method (10,000 bootstrap replications) in MEGA X software (Kumar et al. 2018) based on the 13 protein coding genes of the Cobitidae family, with species of genera *Barbatula* and *Sinogastromyzon* as the outgroups (Figure 1). The phylogenetic tree includes clades 1 and 2, and clade 1 is classified into species of the genus *Misgurnus*, *Cobitis*, *Kichulchoia*, *Iksookimia*, and *Niwaella*. Clade 1 did not form a single lineage due to the presence of species belonging to the genus *Misgurnus*, which experienced mitochondrial introgression. Additionally, in clade 1, *Cobitis*, *Kichulchoia*, *Iksookimia*, and *Niwaella* did not form a single lineage as in previous studies (Park et al. 2018), presumably due to mitochondrial introgression (Kwan et al. 2019). Phylogenetic analyses based on nuclear genes are needed to create a single phylogenetic tree of the Cobitidae (Kwan et al. 2018). The genus *Koreocobitis* of clade 2 formed a strong single lineage, whereas the genus *Misgurnus* did not form a single lineage due to the presence of some species belonging to the genus *Paramisgurnus*. According to Šlechtová et al. (2008), *Misgurnus* and *Paramisgurnus* phylogeny should be studied by investigating mitochondrial introgression following intergenus hybrid formation. In the phylogenetic tree, *K. brevifasciata* is the closest related species to *K. multifasciata*, and the mitochondrial genome of *K. brevifasciata* is expected to provide an important genetic resource for molecular phylogenetic and conservation studies.

**Figure 1.**
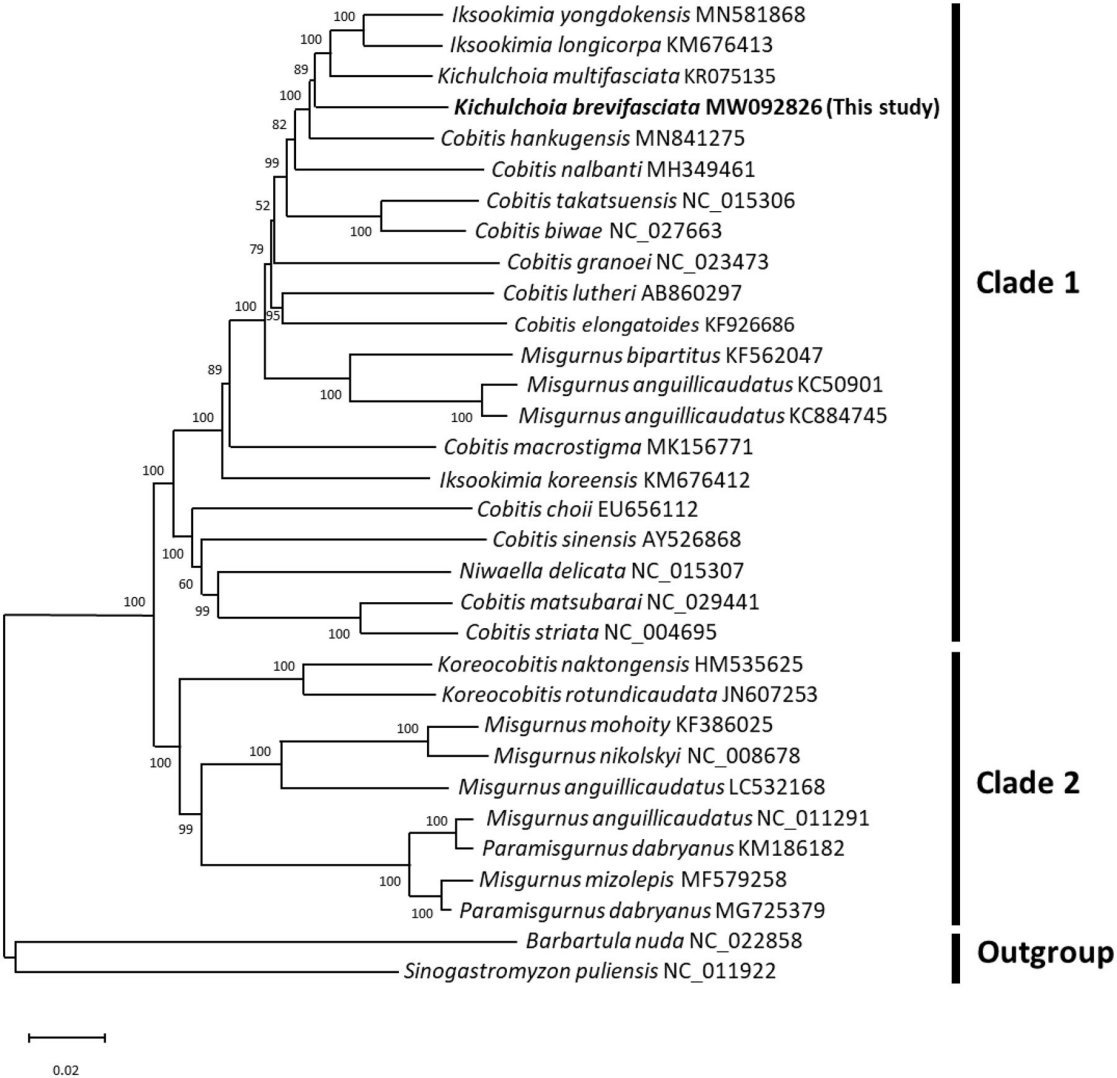
Neighbor-Joining tree of 13 protein coding genes based on Cobitidae species. The bootstrap was repeated 10,000 by the Neighbor-Joining method. GenBank accession number is indicated after the scientific name. The number of each node indicates the probability of a bootstrap support value.

## Data Availability

The genome sequence data that support the findings of this study are openly available in GenBank of NCBI at (https://www.ncbi.nlm.nih.gov/) under the accession no. MW092826. The associated BioProject, SRA, and Bio-Sample numbers are PRJNA687292, SRX9720364 and SAMN17141013, respectively.
